# Influence of EGF and pro-NGF on EGFR/SORTILIN interaction and clinical impact in head and neck squamous cell carcinoma

**DOI:** 10.3389/fonc.2023.661775

**Published:** 2023-07-27

**Authors:** Martin Morisse, Thomas Bourhis, Romain Lévêque, Mathieu Guilbert, Julien Cicero, Martine Palma, Dominique Chevalier, Xuefen le Bourhis, Robert-Alain Toillon, Francois Mouawad

**Affiliations:** ^1^ Department of Otorhinolaryngology and Head and Neck Surgery, University Hospital Center (CHU) de Lille, University of Lille, Lille, France; ^2^ Univ. Lille, Inserm, University Hospital Center (CHU) Lille, UMR9020-U1277 - CANTHER – Cancer Heterogeneity Plasticity and Resistance to Therapies, Lille, France

**Keywords:** HNSCC, EGFR, Sortilin, pro-NGF, NGF, survival

## Abstract

Head and Neck Squamous Cell Carcinoma (HNSCC) remains a cancer with a poor prognosis, with a 5-year survival rate of less than 50%. Although epidermal growth factor receptor (EGFR) is almost always overexpressed, targeted anti-EGFR therapies have modest efficacy and are mainly used in palliative care. Growth factors such as Nerve Growth Factor (NGF) and its precursor proNGF have been shown in our laboratory to play a role in tumor growth and aggressiveness. Interestingly, an interaction between Sortilin, a proNGF receptor, and EGFR has been observed. This interaction appears to interfere with the pro-oncogenic signaling of EGF and modulate the membrane expression of EGFR. The aim of this study was to characterize this interaction biologically, to assess its impact on clinical prognosis and to analyze its role in the cellular trafficking of EGFR. Using immunohistochemical staining on tumor sections from patients treated at our university center and PLA (Proximity Ligation Assay) labeling, we showed that Sortilin expression is significantly associated with reduced 5-year survival. However, when Sortilin was associated with EGFR, this association was not found. Using the Cal-27 and Cal-33 cancer cell lines, we observed that proNGF reduces the effects of EGF on cell growth by inducing the internalization of its receptor. These results therefore suggest a regulatory role for Sortilin in the degradation or renewal of EGFR on the membrane. It would be interesting in future work to show the intracellular fate of EGFR and the role of (pro)neurotrophins in these mechanisms.

## Introduction

Head and neck cancer accounts for approximately 650,000 or nearly 6% of new cancer cases and nearly 350,000 deaths worldwide each year. Head and neck cancers are a diverse group of malignancies that arise in the oral cavity, oropharynx, larynx and hypopharynx. Five-year survival rates for HNSCC patients remain below 50%. Locoregional failure is responsible for the vast majority of deaths from HNSCC (1).

Overexpression, mutation or aberrant activation of tyrosine kinase receptors (TKRs) has been implicated in many diseases, including cancer. EGFR is a member of the human epidermal growth factor receptor (EGFR or HER) tyrosine kinase family, which also includes ErbB2 (HER2/Neu), ErbB3 (HER3) and ErbB4 (HER4). Studies have shown that 90-100% of HNSCCs overexpress EGFR. It is actively involved in the carcinogenesis of these tumors (2). This overexpression occurs early in the multi-step carcinogenesis process of HNSCC, is strongly present in the oral mucosa of smokers, and the level of ligands to EGFR is also increased, allowing an autocrine activation loop (3, 4). It is important to note that despite the overexpression of EGFR in HNSCCs, the response rate to cetuximab as a monotherapy in the treatment of recurrent or metastatic disease is approximately 10-15%. In addition, no factor has been shown to predict response to cetuximab other than the intensity of the rash it may cause (5). EGFR as a prognostic biomarker in HNSCC has been evaluated in several studies. The oldest studies, based on semi-quantitative visual interpretation of immunohistochemical staining, are inconsistent, but more recently quantitative analysis techniques have correlated EGFR overexpression with decreased overall survival in HNSCC and an excess risk of locoregional recurrence after treatment with surgery and radiotherapy (6). Its benefit has also been noted in patients treated with radiotherapy alone: high expression correlates with poorer locoregional control and a significant decrease in overall survival (7). With regard to the response to anti-EGFRs, mainly cetuximab, the available studies do not allow the identification of EGFR expression or EGFR copy number increase as predictive biomarkers to improve the efficacy of this targeted therapy.

**Figure 1:**
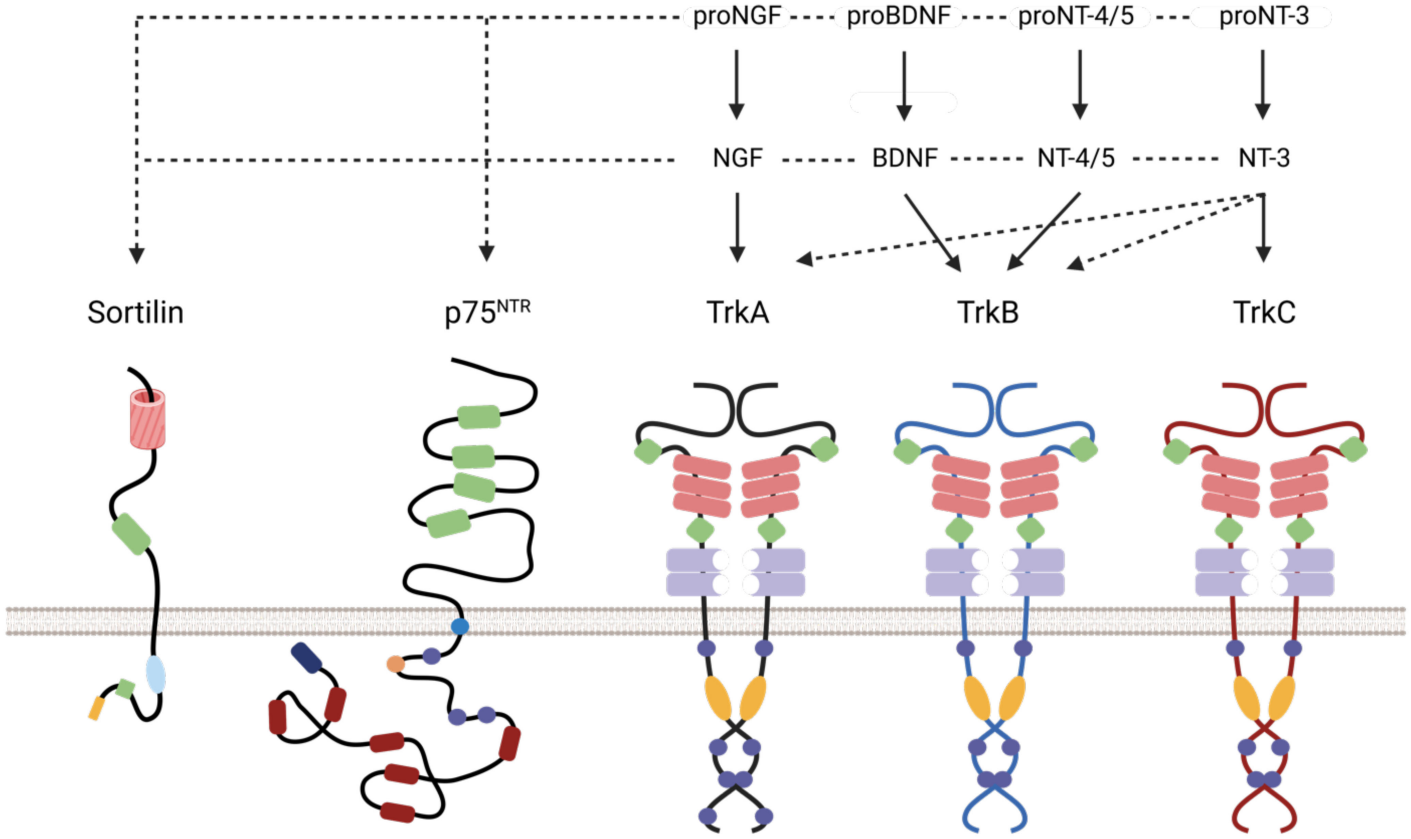
Schematic representation of Neurotrophin receptors/ligand interaction and duolink reaction. Association of different neurotrophins and pro-neurotrophins with their receptors. The pro-neurotrophins interact with Sortilin and the neurotrophins with p75NTR. TrkA is the high affinity receptor for NGF and its precursor, TrkB for BDNF, pro-BDNF and (pro-) NT-4/5, and TrkC for NT-3 and its precursor. The receptors have different extracellular functional domains (cysteine-rich domain (green), leucine-rich domain (red rectangle), Ig-C2 (purple), β-propeller (red cylinder) and intracellular (phosphorylation site (round purple), palmitoylation site (orange circle), tyrosine kinase (orange), death domain (red)).

Peri-nerve growth is associated with an increased risk of loco-regional recurrence, a decrease in disease-free survival (DFS) and a deterioration in quality of life (QOL) with an increase in pain. Perineural growth is explained not only by reduced resistance close to the nerves, but also by the role of the perineural microenvironment in promoting an increase in proliferation and a decrease in apoptosis of cancer cells. Several molecules have been identified as playing a paracrine role in this process, such as BDNF (Brain-Derived Neurotrophic Factor), NT3-4 (Neurotrophins 3 and 4), GDNF (Glial Cell Line Derived Neurotrophic Factor), NCAM (Neural Cell Adhesion Molecule) and NGF (Nerve Growth Factor) (1). Each neurotrophin has a high affinity receptor (TrkA for NGF) and all neurotrophins bind to a common low affinity receptor: p75NTR (a pan-neurotrophin receptor), which does not contain an effector kinase domain. In addition to their role in neuronal growth, neurotrophins are involved in oncogenesis (**Figure 1**). In breast cancer, NGF binding to TrkA promotes proliferation, cell survival, angiogenesis and metastasis (8). Overexpression of NGF in HNSCCs correlates with peri-nerve sheathing and is a marker of poor prognosis in tongue squamous cell carcinoma (9, 10). ProNGF is the precursor of NGF, has a lower affinity than NGF for TrkA and binds mainly to p75NTR and Sortilin. Sortilin (or NTR3 for Neurotensin Receptor 3) belongs to a family of small proteins characterized by the presence of a VPS10 (Vacuolar Protein Sorting 10) domain. Its natural ligand is neurotensin, but through this domain it can also bind to proneurotrophins such as pro-NGF and proBDNF. The location and functions of this protein are diverse. At the membrane level, the primary structure of Sortilin, and in particular the small size of its cytoplasmic carboxy-terminal tail, seems to prevent it from transmitting an intracellular signal on its own. The search for its activities at the cell surface therefore involves identifying partnerships with other membrane proteins, which is the subject of this study. However, membrane expression represents only about 10% of the cellular content of Sortilin, the remaining proteins being located inside the cell. In particular, at the level of the Golgi apparatus (GGA). This intracellular fraction of about 90% is responsible for another function of Sortilin: the regulation of intracellular traffic (11). In particular, it has a cytoplasmic domain with similarities to the mannose-6-phosphate receptor, which interacts with GGA proteins (Golgi-localized, gamma-heart-containing, ADP-ribosylation factor-binding), adaptive proteins that allow the recruitment of clathrin and play a role in endosome maturation and lysosome targeting (12). The importance of proNGF has been highlighted at the neuronal level, where it promotes apoptosis *via* the p75NTR-Sortilin complex, in contrast to NGF, which stimulates cell survival and differentiation *via* TrkA or p75NTR (13). It is involved in many neurodegenerative diseases, but recent studies have also demonstrated its pro-oncogenic role: in breast cancer, pro-NGF increases the invasive potential of cells by interacting with the TrkA/Sortilin complex, independently of p75NTR (14). It is overexpressed in thyroid cancer and has been suggested to play a pro-oncogenic role in pancreatic adenocarcinoma (15, 16). The level of neurotrophin expression in healthy organs correlates with the degree of sympathetic innervation: in the brain, NGF is expressed 20 times more than in other organs. However, it has been shown that cancerous tumors can also secrete neurotrophins, such as prostate and breast cancer, which produce NGF (when it is not found in normal breast epithelial cells), inducing an autocrine loop of activation of the TrkA receptor (17). EGFR signaling is triggered by binding to growth factors such as EGF, which induces autophosphorylation of tyrosine residues on its cytoplasmic domains and recruitment of underlying intracellular signaling pathways (Ras-MAPK-ERK1/2 pathway, Akt/PI3K pathway, Jak/Stat pathway) that promote proliferation, cell survival, migration, invasion, differentiation and angiogenesis. The signaling network downstream of EGFR is one of the most frequently deregulated in cancer, and due to the multiple facets of EGFR cell trafficking, the role of sorting proteins such as Sortilin is of increasing interest. Following phosphorylation, EGFR has been shown to be internalized and both endocytosis and fate of EGFR are then regulated by adapter proteins interacting with the tyrosine kinase domain (18, 19). The rapid internalization of EGFR is a form of negative feedback mechanism of the receptor, involved in limiting the signal induced by EGFR and counterbalancing its pro-oncogenic role. The inactivation of these adaptor proteins, which regulate both the duration and intensity of EGFR signaling, plays an important role in tumor proliferation.

In addition to the known interactions of Sortilin with p75NTR and TrkA, we suspected that Sortilin and one of these ligands, proNGF, interfere with EGFR signaling. The importance of EGFR in HNSCCs and recent work demonstrating EGFR/Sortilin interaction under EGF in bronchial adenocarcinoma led us in this direction (20).

The objectives of this study are first to assess the expression of these different protein players in HNSCC cell lines and to highlight a link with patient prognosis. The role of Sortilin as a positive or negative prognostic factor is indeed ambiguous in the literature. We have characterized the role of proneurotrophins, proNGF, in the signaling, internalization and intracellular fate of EGFR.

## Materials and methods

### Cell lines

The Cal-27 cell line (ATCC^®^, CRL-2095) is the result of a moderately differentiated squamous cell carcinoma of the tongue from an untreated 56-year-old Caucasian man. The Cal-33 cell line (Creative BioArray^®^, CSC-C0479) is the result of a poorly differentiated squamous cell carcinoma of the tongue from a 69-year-old untreated Caucasian male. Cells were cultured in DMEM medium (Gibco^®^, ref. 42430-025) supplemented with 10% FBS (Fetal Bovine Serum), 40 IU/ml penicillin, 40 μg/ml streptomycin, 1% non-essential amino acids and 40 μg/ml gentamicin. To ensure phenotypic homogeneity of cell populations, studies were performed on cells that have undergone a maximum of 20 passages.

### Tissue microarray (TMA) and immunohistochemistry (IHC) on TMA slides

The TMA slides contained 96 head and neck tumor samples (20 benign and 28 malignant tumors, including 11 squamous cell carcinomas), each tumor being divided into 2 samples on the slide (BioChain^®^, Cat. Z7020051, lot B508149). They allowed biological labelling in a single step and good comparability between tumors (homogeneity of labelling). For each tumor, some clinical informations were provided: age and sex of the patient, histology, anatomical location and TNM stage for cancers. The TMA sections were first dewaxed and rehydrated through successive baths of butanol (2x4h), xylene 100% (2x5min), ethanol 100% (2x5min), ethanol 96% (2x5min), ethanol 70% (2x5min). Endogenous peroxidases were blocked with a solution containing 50% methanol, 3% H_2_O_2_ and 47% demineralized water (10 min, 20°C). After two washes with Tris Buffer Saline (TBS) and protein saturation (TBS, 20% FBS, 1h, 20°C), the slides were exposed to the primary antibody diluted in demineralized water containing 1% FBS (16h, 4°C). After washing with PBS (2x5min) and TBS- 0.1% Tween 20 (TBS-T; 2x10min), the slides were exposed to the secondary antibody diluted in demineralized water containing 1% FBS (1h30, 20°C). Revelation was performed using the SIGMA FAST TM kit (D4168-50SET, SIGMA^®^). Counterstaining was carried out in Mayer’s hematoxylin solution. Dehydration was achieved by successive baths of ethanol (70%, 96%, 100%) and butanol.

### HNSCC patient tissue samples

For this study, we used a cohort of 55 patients who have not objected to the use of their data (biological material) and the scientific processing of their medical records. Serial paraffin tumor sections (10 consecutive sections) were performed from patients who underwent surgery for squamous cell carcinoma of the oral cavity or oropharynx at the University Department between 2008 and 2014. Tumor slides were deparaffinized and rehydrated as described above. Antigen unmasking was performed by incubation in a Tris-EDTA (10 mM Tris base, 1 mM EDTA, 0.05% Tween 20 (v/v)) (70°C, 10 min).

*IHC staining*. Endogenous peroxidases were blocked with a solution containing 50% methanol, 3% H_2_O_2_ and 47% demineralized water (10 min, 20°C). After two washes with TBS and protein saturation (TBS-T + FBS 20%, 1h, 20°C), the slides were exposed to the primary antibody diluted in demineralized water containing 1% FBS (16h, 4°C). After washing with PBS (2x5min) and TBS-T (2x10min), the slides were placed in the presence of secondary antibodies coupled to HRP diluted in demineralized water containing 1% FBS (1h30, 20°C). The detection was performed using the SIGMA FAST TM kit (D4168-50SET, SIGMA^®^). Counterstaining was carried out in Mayer’s hematoxylin solution. Dehydration was achieved by successive baths of ethanol (70%, 96%, 100%) and xylene. Images were taken with an evos M5000 microscope (Thermo Fisher scientific).

*PLA staining.* Endogenous peroxidases were blocked using the solution provided in the Duolink Brightfield Kit (Sigma-Aldrich) (10 min, 20°C). After two washes with TBS and protein saturation, the tumors were placed in the presence of the two primary antibodies diluted in the saturation solution of the kit (16h, 4°C). The tumor samples were then incubated with the ‘Plus’ (anti-rabbit) and ‘Minus’ (anti-goat) probes, diluted 1/5 in the antibody dilution solution provided in the kit. The ligation and amplification steps were carried out as recommended by the manufacturer, Sigma-Aldrich.

### Analysis of patient tumor sections and statistics

The computerized medical records of the patients were studied: information on the tumor and risk factors, data on survival and recurrence were collected. Blind interpretation of the slides after labelling was performed by a pathologist for simple immunohistochemical staining and by an independent observer in the laboratory for PLA labelling. Based on these observations, the tumors were divided into 2 groups: absent/weak labelling versus strong labelling. Statistical analyses of survival were performed using R Studio software, in particular the functions survfit (Kaplan-Meier estimator) and survdiff (log-rank test for comparison of survival curves), with p<0.05 considered significant.

### SiRNA transfection

500 µL of 10% FBS medium, 20 µL of Interferin™ and 20 µM siRNA (siGFP=siCTRL, siSort) were incubated (20 min, 20°C). Meanwhile, the seeded cells (Petri dish diameter 100mm) were washed with 5 mL of medium and replenished with 9.5 mL of 10% SVF EMEM medium, then the transfection mixture was applied evenly over the entire surface of the dish (diameter 100, Greiner Bio-One) and the cells were incubated for 48 h (5% CO2, 37°C). The siRNA sequences used were against Sortilin (Eurogentec): CUCUGCUGUUAACACCACCTT compared to control (siGFP) GAUGAACUUCAGGGUCAGCTT.

### RNA extraction and RT-qPCR

Total RNA was isolated from Cal27 cells using the TRIzol reagent (Invitrogen) as suggested by the manufacturer. One µg RNA was reverse transcribed using SensiFAST cDNA Synthesis Kit (Ozyme, BIO-65054) according to the manufacturer’s recommendations. Quantitative PCR was performed with 1 µl of reversely transcribed RNA in a total volume of 10 µL using ONE Green FAST qPCR Premix (Ozyme, OZYA008). The primer sequences used in this study were for: Sortilin 1 (Forward 5’- CCGTCCTATCAATGTGATTAAG-3’; Reverse 5’-CCATATGGTATAGTCCTTCTC-3’) and GAPDH (Forward 5’-AGCCACATCGCTCAGACAC-3’; Reverse 5’-GCCCAATACGACCAAATCC-3’). Relative gene quantification was normalized to GAPDH levels.

### Western blotting

Proteins from cell lysates were separated by SDS-PAGE and transferred to PVDF membranes (GE Whatmann, United Kingdom). The membranes were blocked with 5% bovine serum albumin (BSA) in TBS-T (w/v) (1h, RT) and immunoblotted (overnight, 4°C). The membranes were washed (5 x 5 min) with TBS-T and probed with horseradish peroxidase-conjugated secondary antibody (Jackson Immunoresearch, Beckman Coulter, France). SuperSignal West Pico Substrate (Thermo Scientific, Belgium). Chemiluminescence was detected using the Fusion FX analyser (Vilber, France). The antibodies used in this study were: anti-actin (A2066, Sigma-Aldrich), anti-EGFR (#2232, Cell Signaling) and anti-Sortilin (# 612101, BD Bioscience).

### Clonogenicity assays

Cells were seeded in 6-well plates (CELLSTAR Greiner Bio-One) at 2000 cells/well in a medium containing 2% FCS. After 24 and 48 hours, the cells were treated according to three groups: a group with 0.5 nM proNGF, a group with 0.3 nM EGF, a group with proNGF and EGF. The control group did not receive any treatment. After 10 days of incubation, the cells were fixed with a paraformaldehyde solution (4% in PBS pH 7.4; 30 min; 20°C) and then stained with crystal violet 0.1%. The colonies were then counted and the wells photographed. Statistical analysis was based on 6 wells per condition.

### PLA on cells

Cells were seeded (15,000 cells/well) onto 8-well compartmentalized slides (055071, LabTek I) previously treated with 98% ethanol + 2% HCl (1h, 20°C) followed by two rinses with PBS. If necessary, collagen coating was performed by incubating 400 μL/well of a sterile collagen solution at 30 μg/ml for 1 h at room temperature before rinsing (Corning), drying at room temperature and storage at 4°C. After incubation for 48 hours at 37°C, the cells were starved for 4 hours and then stimulated in the presence of growth factors (proNGF 0.5 nM; EGF 0.3 nM). They were fixed with a solution of paraformaldehyde (4% in PBS; pH 7.4; 30 min; 20°C). Saturation is performed with a solution of PBS + 4% BSA (1h, 20°C). Incubation with the two primary antibodies was performed without a permeabilization step in a solution of PBS + 4% BSA (16h, 4°C, with shaking). The cells were then incubated with the ‘Plus’ (anti-rabbit) and ‘Minus’ (anti-goat) probes from the Duolink kit (Sigma-Aldrich^®^), diluted 1/5 in PBS + 4% BSA (1h, 37°C). The ligation and amplification steps were carried out according to the manufacturer’s recommendations (Sigma-Aldrich). Nuclei were stained by incubation with Hoechst 33258 (1 mM, 10 min, 20°C, in the dark). Images were taken under a fluorescence microscope (Eclipse TiU, Nikon, 100x oil objective). Thirty fields were photographed randomly per condition. The points representing the PLA signal were counted using Image J^®^ software and in-house plug-in. The comparison of the mean of the red dots per cell was made by Student’s T-test after checking the normality of the distribution of the samples.

### Immunofluorescence and colocalization assay

Cells were cultured for 48 hours (15,000 cells/well) on 8-well compartmentalized slides (055071, LabTek I) with a prior collagen ‘coating’. After starvation and stimulation (proNGF 0.5 nM; EGF 0.3 nM), they were fixed with a 4% paraformaldehyde solution. Cells were permeabilized with a solution of PBS + 0.3% Triton X-100 (2 x 5 min, 20°C) and saturation was done with a solution of PBS + 4% BSA + 0.3% Triton X-100 (1 h, 20°C). Then cells were incubated with primary antibodies in PBS + 4% BSA solution (16h, 4°C, with shaking). After rinsing with PBS (3 x 10 min, 20°C), incubation with labelled secondary antibodies was performed (1h30, 20°C, dark). The nuclei were stained by incubation with Hoechst 33258 (1mM, 30min, 20°C, darkness). After checking the staining by conventional microscopy, the slides were analyzed by confocal laser scanning microscopy (ZEISS^®^ LSM 880). Ten fields per condition were randomly selected and examined; the section plane was chosen to be visually close to the ‘equator’ of the nucleus. For colocalization analysis, the weighted overlap coefficient of Manders (Weighted Colocalization Coefficients) was obtained by image processing with Zen Black 2.3 software (ZEISS^®^). In order not to take into account the autofluorescence of the cells and to fix the cursor on the colocalization graph *a priori*, the unmarked and mono-labelled control samples were analyzed beforehand with the same microscopic settings. This coefficient was compared for each condition.

All the antibodies used in this study and the conditions of use are reported in **Supplementary Table 1**.

## Results

### Histological detection of EGFR, Sortilin and pro-NGF in HNSCC

**Figure 2:**
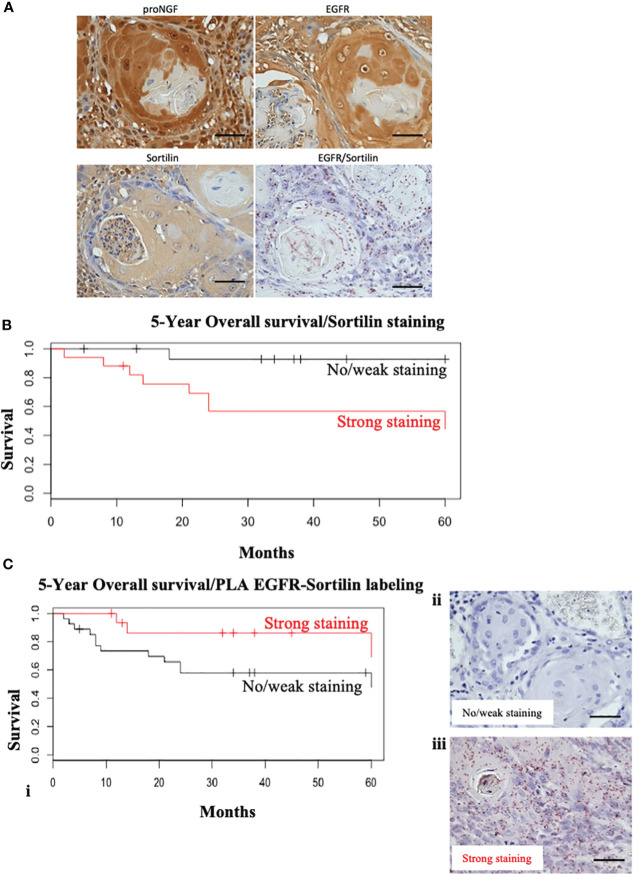
Sortilin decreases overall survival in HNSCC but nor EGFR/Sortilin complexes. **(A)** Examples of labeling performed on TMA slides. Two tumors with 4 different stains were compared: simple IHC staining for proNGF, Sortilin and EGFR and Duolink Brightfield^®^ PLA staining for EGFR/Sortilin interaction. Photographs taken under EVOS M5000 light microscope (x20). **(B)** Kaplan-Meier survival curve over 5 years as a function of the intensity of HRP immunohistochemical stainin of Sortilin (in red = strong staining; in black = weak or absent staining). The vertical lines represent the censored data (patients lost to follow-up or follow-up less than 5 years). **(C)** (i) Kaplan-Meier 5-year overall survival curve as a function of Duolink Brightfield EGFR/Sortiline PLA labeling intensity (in red = strong labeling; in black = weak or absent labeling). The vertical lines represent the censored data (patients lost to follow-up or with less than 5 years of follow-up). Example of light microscopy (x20) of light/no (ii) and strong (iii) PLA EGFR/sortilin staining (acquisition by EVOS M5000 microscope). Scale bar: 50 μm.

Stainings for EGFR, Sortilin and pro-NGF were performed as well as PLA-EGFR/Sortilin staining on HNSCC TMA slides (**Figure 2A**). All HNSCC samples expressed high levels of EGFR (**Figure 2A**), in line with literature data (90-100% EGFR expression in HNSCC). All tumors also expressed proNGF and Sortilin (a ubiquitous protein in human tissue) (**Figure 2A**). In addition, there was a heterogeneity in EGFR/Sortilin PLA labelling, which does not appear to correlate with simple labelling of proNGF, EGFR or Sortilin (**Figure 2A**).

### Correlation between Sortilin staining and overall survival

Immunohistochemical staining was then performed on tumor sections from a cohort of 55 patients from our university department (**Figures 2B, C**). The level of EGFR expression and its association with prognosis have been evaluated in several studies (5, 21, 22). Therefore, we decided to focus on the expression of Sortilin and the EGFR/Sortilin pair in HNSCCs. For Sortilin, 52 out of 55 slides could be stained and/or analyzed. The slides were divided into 2 groups according to the intensity of the staining: “weak to moderate” and “strong to intense” by an independent pathologist (26 slides in each group; three slides were useless). Correlation between staining and tumor characteristics (**Table 1**) or patient prognosis was then assessed (**Figures 2B, C**). The Kaplan-Meier curve analyzing overall survival at 5 years showed a significant decrease in overall survival (log-rank=4, p=0.0445) for strong Sortilin staining in tumors (**Figure 2B**). This trend was not seen when comparing relapse-free survival between these two groups (log-rank=0.6, p=0.417). EGFR/Sortilin complexes were then analyzed by proximity ligation assay and divided into two groups (**Figure 2C**). Thirty-three slides showed strong PLA labelling (**Figure 2C** iii) and 19 showed weak labelling (**Figure 2C** ii). The Kaplan-Meier curve analyzing overall survival at 5 years showed a trend towards increased overall survival with strong EGFR/Sortilin interaction in tumors (**Figure 2C** i). However, this trend was not statistically significant when comparing the two curves using the log-rank test (p=0.055). This non-significant trend was seen when analyzing 5-year relapse-free survival (p=0.07).

**Table 1:** Characteristics of the patient cohort studied, their tumors and pathology progression.

			Sortilin staining				EGFR/Sortilin staining		
			Weak to moderate	High	Not determined	P value	Wek to moderate	High	P value
**N =**		55	26	26	3		35	20	
**Genre**	**female**	16	11	4	1	0.09667	9	7	0.8112
	**male**	39	16	21	2		25	14	
**Age**	**mean**	56.7 yrs				0.7954			0.2044
	**>/= 60 years**	22	12	9	1		14	8	
	**< 60 years**	33	14	17	2		21	12	
**Tobacco**	**yes**	47	23	21	3	0.7007	27	20	0.2626
	**no**	8	3	5	0		7	1	
**Tumor**	**T1**	2	2	0	0	0.4020	1	1	0.2648
	**T2**	21	10	11	0		11	10	
	**T3**	17	8	6	3		14	3	
	**T4a**	15	6	9	0		9	6	
**Stage**	**I**	2	2	0	0	0.2378	1	1	0.6071
	**II**	7	2	5	0		4	3	
	**III**	12	5	4	3		10	2	
	**IVa**	32	17	15	0		19	13	
	**IVb**	2	0	2	0		1	1	
**localization**	**oral cavity**	36	16	18	2	0.7707	22	14	0.8094
	**oropharynx**	19	10	8	1		13	6	
**Relapse**	**Total**	26	10	14	2	0.4040	20	6	0.09715
	**T**	20	8	11	1	0.5646	14	6	0.6525
	**N**	11	4	7	0	0.4971	8	3	0.7261
	**M**	5	3	1	1	0.6594	4	1	0.6929
**PNI positive**		22	13	7	2	0.1541	14	8	1
**vascular emboli positive**		18	7	9	2	0.7638	13	5	0.5323
**Lymph node positive**		29	17	11	1	0.1643	15	14	0.1715
**capsular rupture**		22	12	9	1	0.5719	14	8	1

Patient and tumor characteristics were compared between subgroups using Chi-square tests (chisq.test) for discrete qualitative or quantitative variables, and Student’s t-tests (t.test) for quantitative variables, applying a first-species risk α of 5%.

### ProNGF interferes with the effects of EGF on HNSCC clonogenicity

**Figure 3:**
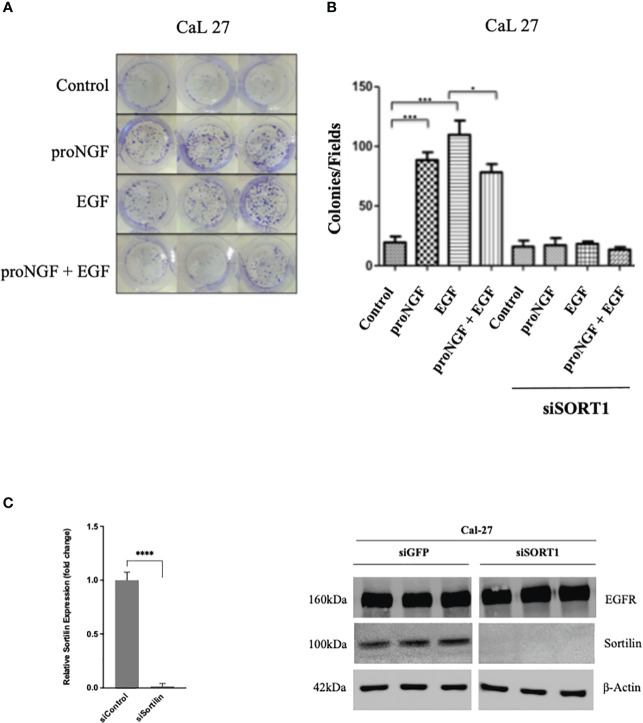
Effects of EGF and proNGF on clonogenic cell growth of CAL 27 cells. **(A)** Example of CAL 27 colony forming units visualized by crystal violet staining. **(B)** Results are the average of 6 wells, based on the number of colonies visible to the naked eye per well. Comparison of means by non-parametric Mann-Whitney test (*p <0.05, **p <0.01, ***p <0.001). 2000 cells per well for Cal 27, 5000 cells per well for siSORT1. **(C)** Histogram representing the difference in relative expression of SORT1 after siSORT1 compared to siGFP, RTqPCR on Cal 27 cell line. Average expression of 3 samples. ****p<0.0001 (Student t-test). Western blot results on Cal 27 cell line after transient transfection with siRNA targeting the expression of SORT1 relative to a siGFP (sicontrol).

To observe the biological effects of EGF and proNGF on HNSCC, clonogenicity assays were performed on Cal 27 cells stimulated with EGF and proNGF (**Figures 3A, B**). Clonogenicity was increased for cells stimulated by EGF and pro-NGF compared to the control. The colonies were also larger indicating that there is an increased proliferation under these conditions. Interestingly, proNGF appeared to interfere with the EGF signaling pathway, with significantly lower clonogenicity when cells were treated simultaneously with both growth factors compared to those stimulated by EGF alone (mean of 73.3 colonies versus 109.6; p = 0.045). In order to confirm Sortilin involvement in clonogenicity induced by proNGF and/or EGF, Sortilin expression was decreased by siRNA (**Figures 3B, C**). Nevertheless, Sortilin siRNA dramatically reduced CAL 27 clonogenicity and did not allow to conclude on the effect of proNGF binding to Sortilin (**Figure 3B**). Control experiments revealed that siRNA efficiently reduced Sortilin expression as shown by qRT-PCR and western blot (**Figure 3C**).

**Figure 4:**
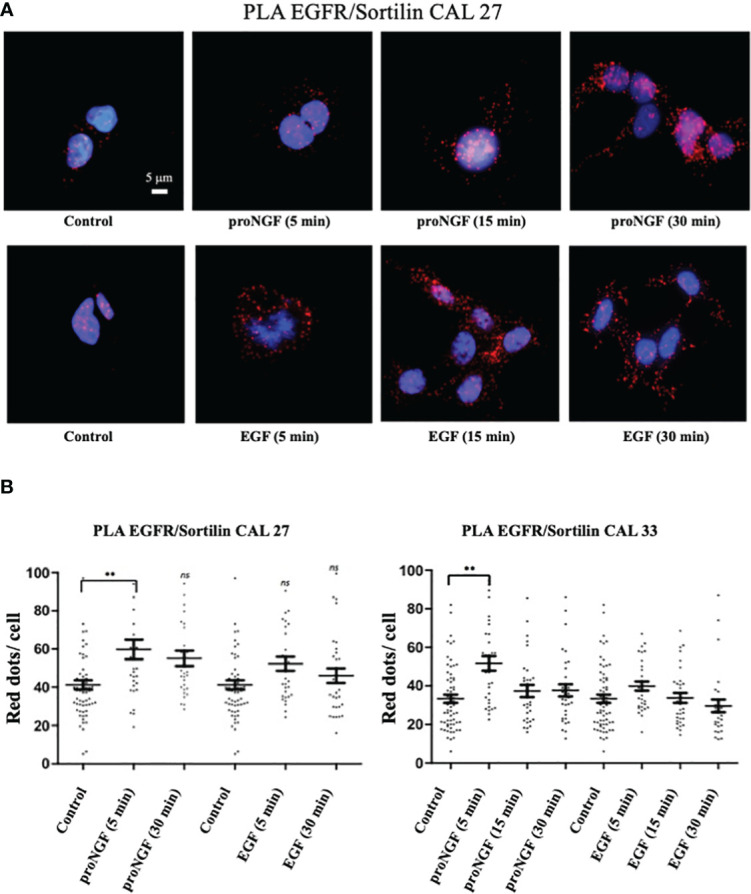
EGFR/sortilin complex formation assessed by PLA. **(A)** EGFR/sortilin PLA results on Cal 27 cells *in vitro* after stimulation with pro-NGF and EGF for 5-, 15- and 30-min. nuclei were labeled with Hoechst 32258 (blue). PLA signal from EGFR/sortilin complexes was visualized as red dots. **(B)** Quantification of EGFR/sortilin PLA signal. Results are expressed as number of complexes per cell (average for each condition over 30 fields examined). Comparison of mean scores per cell was based on a parametric ANOVA test after checking the normality of the sample distribution, followed by a Bonferroni post-test (*p<0.05, **p<0.01, ***p<0.001, ns, not significant).

*EGF and pro-NGF promote EGFR/Sortilin membrane interaction.* As previously described, Sortilin was involved in several pathways between the plasma membrane, endosomes, the trans-Golgi network and lysosomes. To study the presence of an EGFR/Sortilin interaction at the level of the plasma membrane in the basal state and under the influence of EGF and proNGF, a proximity ligation assays (PLA) were performed on Cal 27 and Cal 33 cells without prior membrane permeabilization (**Figure 4**). We observed the presence of a significant EGFR-Sortilin interaction in the basal state with an average of 20 to 40 dots/cell, depending on the experiment as showed for CAL 27 cells (**Figure 4A**). proNGF Stimulation favored a significant increase in this interaction, with an overall doubling of the number of points per cell after 5 min of stimulation both in CAL 27 and CAL 33 cells (**Figure 4B**). In addition, we observed that the formation of this membrane complex was not significantly increased with EGF neither at 5 min nor 30 min of treatment (**Figure 4B**).

### EGF and pro-NGF promote endocytosis and the presence of EGFR in early endosomes

**Figure 5:**
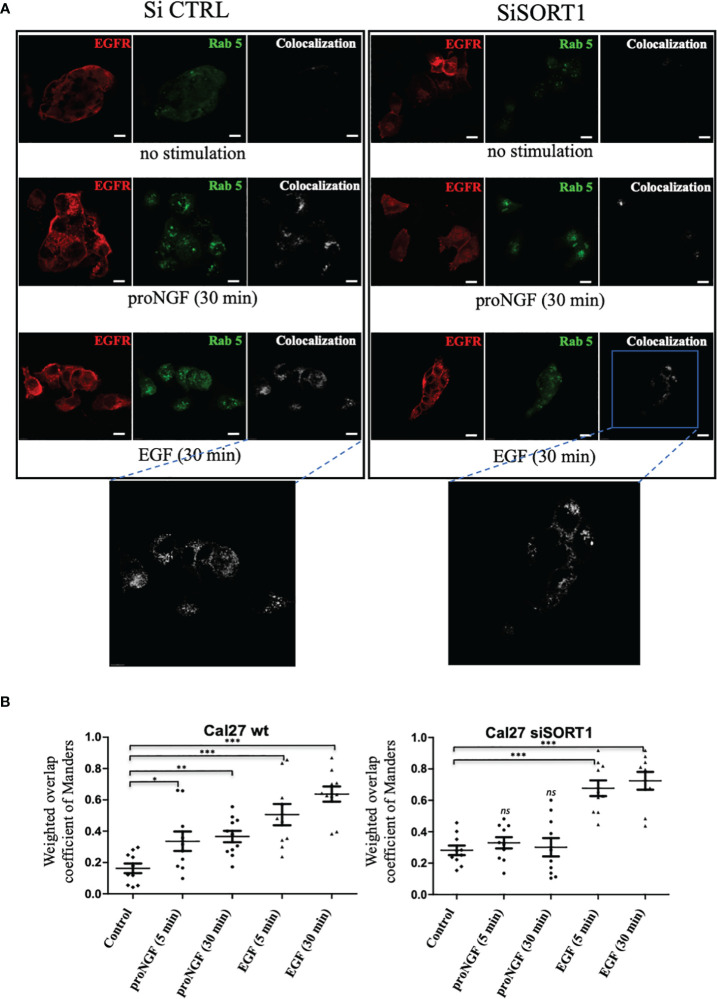
proNGF induced EGFR internalization is dependent of sortilin. **(A)** Colocalization of EGFR/Rab5 assessed by immunofluorescence. The confocal laser scanning microscope settings were maintained between all conditions. EGFR/Rab5 colocalization was assessed on Cal 27 wt and Cal 27 cells with inhibition of SORT1 expression by siRNA. Green fluorochrome corresponded to EGFR (AlexaFluor 488), red to Rab5 (AlexaFluor 546) and white to the colocalization channel. **(B)** The scatter plots showed the results of the calculation of the Manders weighted overlap coefficient for the red channel in each condition (10 images coefficients are compared using a non-parametric Mann-Whitney test (*p<0.05, **p<0.01, ***p<0.001, ns, not significant)).

As proNGF induced EGFR Sortilin complex formation, the role of Sortilin was then assessed on EGFR endocytosis under proNGF or EGF treatment (**Figure 5**). Rab5 was used as an early endosome marker (23–25). By colocalization experiments, we observed that EGFR is colocalized with Rab5 in the absence of stimulation (basal level). Moreover, both proNGF and EGF treatments increased EGFR/Rab5 colocalization (at 5 and 30 min of treatment) indicating that proNGF and EGF induce EGFR endocytosis. Indeed, it has been described in the literature that inactivated EGFR can be endocytosed *via* preformed clathrin-coated wells and that phosphorylation of this receptor accelerates this endocytosis by recruiting the adaptor protein AP2, which promotes the faster organization of clathrin-coated vesicles (26). The extension of this colocalization under EGF to 5 minutes also implies the use of a “fast” endocytosis independent of clathrin. This would involve the ubiquitination of EGFR by the ubiquitin ligase Cbl, promoted by the adaptor protein Grb2 (27, 28). As these elements are known from the literature, the most interesting result here was the significant increase in EGFR-Rab5 colocalization after stimulation with proNGF. This suggested a role for Sortilin in the endocytosis of proneurotrophin-dependent EGFR (**Figure 5**). So, using siSORT1, the effect of invalidation of Sortilin was assessed on EGFR endocytosis. Indeed, Sortilin inhibition decreased EGFR/Rab5 co-localization under proNGF at 5 and 30 minutes (**Figure 5B**), since there is no longer a significant difference with the control condition. By contrast, Sortilin invalidation did not decrease EGFR/Rab5 colocalization indicating that EGFR endocytosis did not involve Sortilin under EGF treatment.

## Discussion

The importance of Sortilin as a receptor or co-receptor for neurotrophins has been demonstrated in the development and function of the nervous system, particularly in the life and survival of neuronal cells (13, 29). Sortilin expression is elevated in many human cell lines and controls the trafficking and release of neurotrophins. Disorders in the autocrine/paracrine loop of neurotrophins, in addition to the interaction of Sortilin with various membrane receptors, appears to be involved in neurodegenerative diseases as well as cancer (30). For example, Sortilin expression is increased in glioma, colon, pancreatic and skin cancers (31–33). In breast cancer, in addition to the overexpression of Sortilin in cancerous tissue compared to healthy tissue, an association between this overexpression and lymph node invasion has been shown (34).

In addition, it has been shown that Sortilin is a key element in the biogenesis of exosomes expressing TrkB and EGFR, which appears to favor angiogenesis within the tumor microenvironment (35). There are currently no studies on the prognostic or predictive role of Sortilin. We show here that in HNSCC, overexpression of this protein appears to correlate with a poorer prognosis in terms of 5-year overall survival.

In contrast, in hormone-refractory prostate cancer, Sortilin is weakly expressed and plays a more protective role by promoting the endocytosis and lysosomal targeting of progranulin, an autocrine growth factor that favors the migration, proliferation and clonogenicity of prostate tumor cells (36). In their article on lung adenocarcinoma, Al-Akhrass et al. identify Sortilin as a key regulator of EGFR cell trafficking, limiting its pro-proliferative signaling (20). Using confocal microscopy, they show that a decrease in Sortilin expression increased the membrane localization of EGFR. They also observe that Sortilin depletion in xenografted mice promoted proliferation and tumor growth. They conclude that in mutant lung cancer cells overexpressing EGFR and resistant to tyrosine kinase inhibitors, Sortilin acts as a tumor suppressor, whereas in other cancers it promotes malignant cell behavior (20). Since HNSCCs overexpress EGFR and are naturally resistant to tyrosine kinase inhibitors, we might expect the same result. However, as we have seen, the functions of Sortilin are not limited to its sorting role. It therefore seemed necessary to look more closely at the role of Sortilin when it interacts with EGFR. Thus, by analyzing PLA staining on tumor sections, we suspected that a strong association between EGFR and Sortilin seemed to improve OS. These data, although not significant, could not support the hypothesis proposed by Al-Akhrass et al. that Sortilin, when overexpressed, improves prognosis through its interaction with EGFR. So, the demonstration of Sortilin and the EGFR/Sortilin interaction as a prognostic or predictive factor requires additional experiments to be validated. In fact, we could not identify any element that could explain the excess mortality in the “strong staining” group. In the patients studied, this staining does not correlate significantly with the other clinical and histological criteria of poor prognosis (tumor size, lymph node invasion, peri-nervous invasion, nodular capsular rupture, incomplete resection) or with the decrease in disease-free survival (DFS).

Using PLA labelling on cells, we were able to demonstrate an increase in the interaction between EGFR and Sortilin in Cal 27 cells under EGF and proNGF. This is the first study to highlight the formation of this complex under EGF in head and neck cancer, following the article on lung adenocarcinoma where this interaction was confirmed by FRET (fluorescence resonance energy transfer) and immunoprecipitation (20). EGFR and Sortilin have also been shown to interact through their extracellular domains. The extracellular domain of Sortilin is called VPS10 (Vacuolar Protein Sorting 10) and is known to interact with numerous proteins that benefit from sorting between the different cellular sub compartments, in particular the trans-Golgi network and the lysosome (37, 38). In PLA, ProNGF, as a ligand for Sortilin, also seems to induce this membrane interaction. This new fact suggests an important regulatory role for this proneurotrophin in the EGFR signaling pathways. The demonstration of the interference of proNGF in the EGF signaling pathway by functional biological assays is particularly interesting in our study. The results of the clonogenicity tests confirm our initial hypothesis. It will be necessary to continue to investigate the role of neurotrophins and their precursors in known pro-oncogenic signaling pathways.

**Figure 6:**
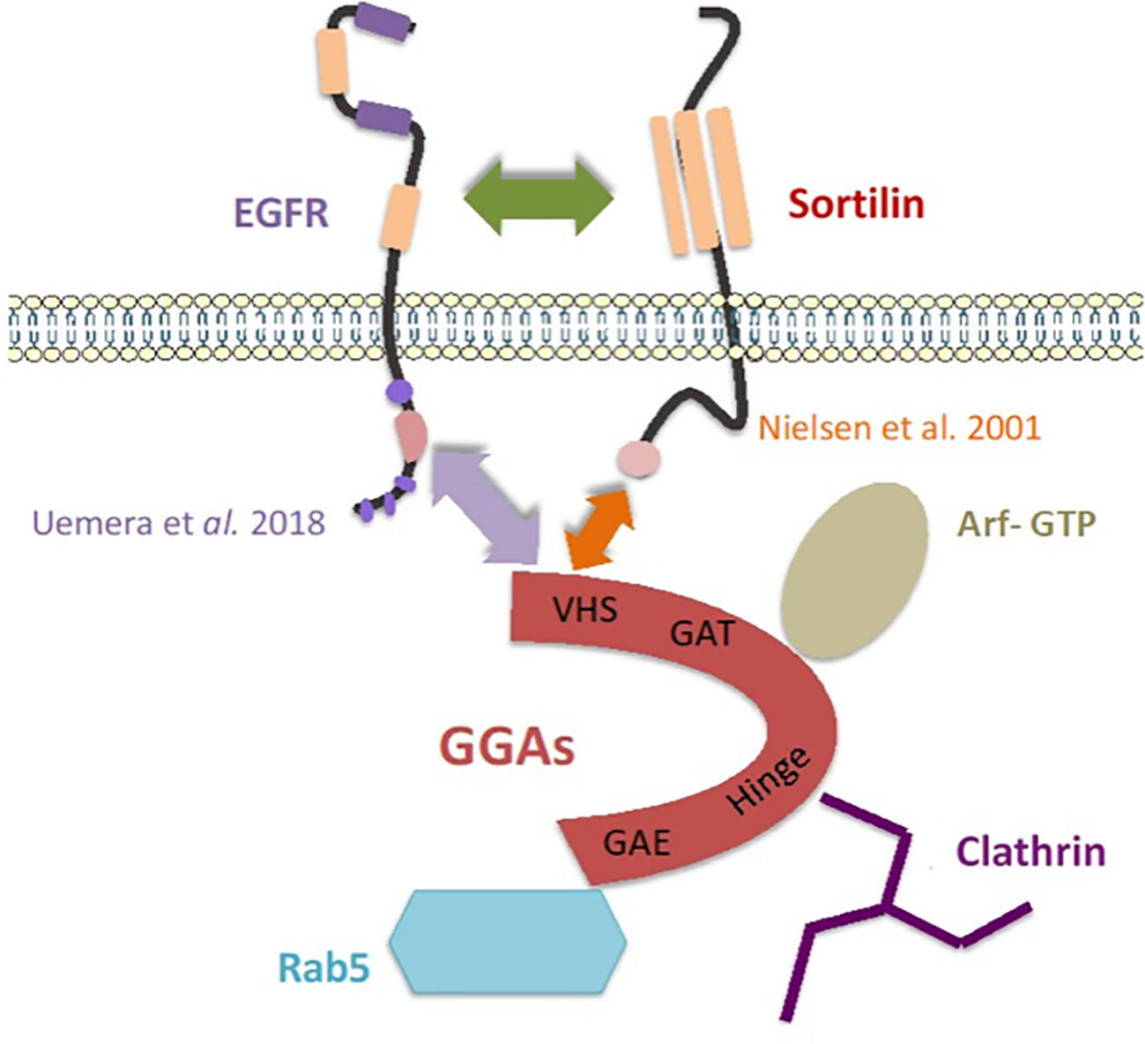
Model of an addressing “cargo ship” involving Sortilin and the GGAs proteins, adapted from Mazella and Vincent (43). The arrows represent the interactions highlighted in the literature and in this work (20, 41, 42).

On the other hand, in breast cancer, the decrease in clonogenicity of cells after siSORT1 has already been observed in the literature. Functional assays on MDA-MB-231 have shown that Sortilin inhibition alters cell proliferation, survival and adhesion *in vitro* (34) and we have shown that a ternary complex between Sortilin/TrkA/EphA2 is involved in breast cancer cells (39). The results of colocalization assays support an internalization of EGFR induced by proNGF and we observed that EGFR endocytosis is decreased when Sortilin expression is inhibited by siSORT1. For EGF, this effect is independent of Sortilin. Hence, the importance of tyrosine 1068 phosphorylation of the tyrosine kinase domain has been demonstrated in this internalization with recruitment of the adaptor protein Grb2 (growth factor receptor-bound protein 2), the AP-2/clathrin complexes and the ubiquitin ligase Cbl (40). Sigismund et al. have studied the fate of EGFR after internalization following endocytosis (26). Clathrin-dependent endocytosis (CDE) is the main mechanism of internalization of EGFR, which is then sorted within early endosomes for membrane recycling or degradation *via* late endosomes. However, there are alternative pathways for the internalization of tyrosine kinase receptors, for example involving caveolin. It has been shown in HeLa cells that the mode of internalization appears to influence the fate of EGFR, with CDE promoting EGFR recycling and signaling, whereas clathrin-independent endocytosis is mainly involved in EGFR degradation. Under the influence of EGF, Sortilin appears to promote clathrin-dependent internalization in lung cancer (20). Therefore, the main goal of our project is to understand the role of proNGF *via* Sortilin in the balance between signaling, degradation and recycling of EGFR. To do this, it could be used the same co-localization techniques described with late endosomal markers (such as Rab7), sorting towards the lysosome and using Dynasore (a dynamin inhibitor that blocks CDE) or by inhibiting clathrin with siRNA. GGA proteins have also recently been shown to be involved in the stabilization (GGA 2) or membrane turnover (GGA 1/3) of EGFR, with the authors highlighting an interaction between the juxta-membrane of EGFR and the VHS domain of GGA proteins (41). It is suggested that Sortilin is involved in this process through its interaction with GGAs (42). These various interactions are summarized in **Figure 6**.

Future studies may be interested in the importance of other Sortilin ligands in HNSCC, such as pro-BDNF. The role of BDNF in perineural growth in HNSCC has been mentioned. It appears to be involved in invasion, metastasis and resistance to chemotherapy *via* its high affinity receptor TrkB (44, 45).

## Conclusion

In this study we confirmed for the first time the presence of EGFR, Sortilin and proNGF in HNSCC tumors. There seems to be a correlation between high expression of Sortilin and decreased overall survival at 5 years, as has been shown in other cancers. However, if overexpression of Sortilin appears to be associated with a poor prognosis, its interaction with EGFR at the membrane level could favor the internalization of EGFR and the reduction of its membrane expression. Finally, we have shown that pro-NGF and EGF promote the formation of this EGFR/Sortilin complex and the internalization of the Sortilin-dependent EGFR complex. However, we do not know the weight of these growth factors in this mechanism, the level of phosphorylation of EGFR at the time of internalization and the fate of intracellular EGFR. In addition, the signaling pathways downstream of Sortilin and its co-receptors that are involved in tumor progression or recurrence remain poorly understood and require further work.

## Data availability statement

The raw data supporting the conclusions of this article will be made available by the authors, without undue reservation.

## Ethics statement

Ethical review and approval were not required for the study on human participants in accordance with the local legislation and institutional requirements. The patients/participants provided their written informed consent to participate in this study.

## Author contributions

Conceptualization: R-AT, FM. Data curation: MG, RL, MM, R-AT. Formal analysis: MM, TB, MG, RL. Funding acquisition: R-AT, XB. Investigation: MM, TB, MG, RL. Methodology: R-AT, FM. Project administration: R-AT, XB. Supervision: MG, RL, R-AT, FM. Validation: R-AT, DC, FM. Visualization: MM, TB, R-AT, FM Roles/Writing – original draft: FM, MM Writing – review & editing: FM, MM, R-AT. All authors contributed to the article and approved the submitted version.
