# Supplementary material for: Influence of EGF and pro-NGF on EGFR/SORTILIN interaction and clinical impact in head and neck squamous cell carcinoma
Source: Front Oncol. 2023 Jul 27;13:661775. doi: 10.3389/fonc.2023.661775 (PMC10416107; doi:10.3389/fonc.2023.661775)
Supplement: Supplementary file 1 [file Table_1.docx]

| **Antibody** | **Reference** | **Species** | **Dilution** |
| --- | --- | --- | --- |
| Anti-EGFR  Anti-P-EGFR | Kit ♯9922,  Cell Signaling Technology | Rabbit | PLA (cells): 1/50 |
| Anti-EGFR | Ref. SC-03,  Santa Cruz Biotechnology | Rabbit | IHC and PLA (tumors): 1/50 |
| Anti-EGFR | Ref. AF231, R&D Systems | Goat | Flow cytometry: 1/80  Immunofluorescence 1/100 |
| Anti-Sortiline | Ref. 612101, BD-Biosciences | Rabbit | Western Blot: 1/500 |
| Anti-Sortiline | Ref. AF3154, R&D Systems | Goat | IHC and PLA: 1/50 |
| Anti-Pro-NGF | Ref. ANT-005, Alomone Labs | Rabbit | IHC : 1/100 |
| Anti-Goat | Alexa Fluor 488 #11055  ThermoFischer Scientific | Donkey | Flow cytometry: 1/2000  Immunofluorescence 1/2000 |
| Anti-Rabbit | Alexa Fluor 546. Invitrogen # A-11010  ThermoFischer Scientific | Goat | Immunofluorescence 1/2000 |
| Anti-EEA1 | EEA1Antibody #2411  Cell Signaling Technology | Rabbit | Immunofluorescence 1/100 |
| Anti-Rab5 | Rab5 (C8B1) mAb #3547  Cell Signaling Technology | Rabbit | Immunofluorescence 1/100 |

**Supplementary Table 1 :** **List and references of antibodies used**
